# Inhibition of Hedgehog Signaling Antagonizes Serous Ovarian Cancer Growth in a Primary Xenograft Model

**DOI:** 10.1371/journal.pone.0028077

**Published:** 2011-11-29

**Authors:** Christopher K. McCann, Whitfield B. Growdon, Kashmira Kulkarni-Datar, Michael D. Curley, Anne M. Friel, Jennifer L. Proctor, Hana Sheikh, Igor Deyneko, Jeanne A. Ferguson, Vinod Vathipadiekal, Michael J. Birrer, Darrell R. Borger, Gayatry Mohapatra, Lawrence R. Zukerberg, Rosemary Foster, John R. MacDougall, Bo R. Rueda

**Affiliations:** 1 Department of Vincent Obstetrics and Gynecology, Vincent Center for Reproductive Biology, Massachusetts General Hospital, Boston, Massachusetts, United States of America; 2 Division of Gynecologic Oncology, Department of Vincent Obstetrics and Gynecology, Massachusetts General Hospital, Boston, Massachusetts, United States of America; 3 Harvard Medical School, Boston, Massachusetts, United States of America; 4 Infinity Pharmaceuticals, Cambridge, Massachusetts, United States of America; 5 Division of Hematology-Oncology, Massachusetts General Hospital Cancer Center, Boston, Massachusetts, United States of America; 6 Department of Pathology, Massachusetts General Hospital, Boston, Massachusetts, United States of America; Cedars-Sinai Medical Center, United States of America

## Abstract

**Background:**

Recent evidence links aberrant activation of Hedgehog (Hh) signaling with the pathogenesis of several cancers including medulloblastoma, basal cell, small cell lung, pancreatic, prostate and ovarian. This investigation was designed to determine if inhibition of this pathway could inhibit serous ovarian cancer growth.

**Methodology:**

We utilized an *in vivo* pre-clinical model of serous ovarian cancer to characterize the anti-tumor activity of Hh pathway inhibitors cyclopamine and a clinically applicable derivative, IPI-926. Primary human serous ovarian tumor tissue was used to generate tumor xenografts in mice that were subsequently treated with cyclopamine or IPI-926.

**Principal Findings:**

Both compounds demonstrated significant anti-tumor activity as single agents. When IPI-926 was used in combination with paclitaxel and carboplatinum (T/C), no synergistic effect was observed, though sustained treatment with IPI-926 after cessation of T/C continued to suppress tumor growth. Hh pathway activity was analyzed by RT-PCR to assess changes in *Gli1* transcript levels. A single dose of IPI-926 inhibited mouse stromal *Gli1* transcript levels at 24 hours with unchanged human intra-tumor *Gli1* levels. Chronic IPI-926 therapy for 21 days, however, inhibited Hh signaling in both mouse stromal and human tumor cells. Expression data from the micro-dissected stroma in human serous ovarian tumors confirmed the presence of *Gli1* transcript and a significant association between elevated *Gli1* transcript levels and worsened survival.

**Conclusions/Significance:**

IPI-926 treatment inhibits serous tumor growth suggesting the Hh signaling pathway contributes to the pathogenesis of ovarian cancer and may hold promise as a novel therapeutic target, especially in the maintenance setting.

## Introduction

In the United States, ovarian cancer is estimated to afflict approximately 22,000 women and cause nearly 14,000 deaths annually. The lifetime risk of developing ovarian cancer is 1 in 70 and it is the fifth most lethal cancer in women [1]. Most ovarian cancer patients present with late-stage disease that is treated with surgical debulking and platinum based chemotherapy. Although 70–80% of women achieve a complete clinical response, a majority of those patients will develop recurrent disease that is frequently chemoresistant. Novel treatment approaches utilizing conventional cytotoxic therapies in combination with molecularly targeted therapies directed against specific signaling pathways required for tumor development and progression potentially hold promise as strategies for durable treatment of primary and recurrent ovarian cancer [2].

The Hedgehog (Hh) signal transduction pathway comprises a family of highly conserved proteins that primarily act during embryogenesis to regulate stem cell fate and organogenesis, and promote proliferation, regeneration and differentiation of somatic tissues in the adult [3]. Patched 1 (Ptch1), a membrane receptor, normally inhibits the membrane protein Smoothened (Smo) from activating Gli1. The binding of Hh ligand (Sonic, Indian or Desert) to Ptch1 abrogates its repressive effects on SMO allowing the translocation of Gli1 to the nucleus where it induces the expression of target genes [4], [5]. Aberrant activation of the Hh pathway in adulthood has been associated with the development of malignant transformation in a variety of human cancers [4], [6], [7], [8], [9], [10], [11], [12]. Additionally, tumor initiating cells in some cancers have been shown to be dependent on sustained Hh induced signaling and subsequent activation of Gli1 resulting from ligand over-expression or mutational activation of the Hh pathway [6], [13]. Treatment regimens with Hh pathway antagonists in combination with conventional molecular and cytotoxic therapies have demonstrated *in vitro* and *in vivo* activity against proliferation in medulloblastoma, basal cell, breast, small cell lung, prostate and pancreatic cancer models [10], [11], [12], [14], [15], [16], [17]. These antagonists are currently in Phase I and Phase II clinical trials.

Activation of the Hh pathway has been documented in ovarian cancer as a potential mechanism involved in neoplasia. Altered gene and protein expression of the Hh pathway members Gli1, Smo, Ptch1, Desert hedgehog (Dhh) and Sonic hedgehog (Shh) in ovarian cancer has been reported, although the exact prevalence and pattern remains to be clarified [18], [19], [20], [21]. While many studies suggest that 50–60% of invasive ovarian tumors manifest Hh pathway activation, other investigators have argued that meaningful activation via altered expression of multiple pathway proteins occurs in less than 20% of clinical samples tested [19], [20]. While a direct correlation between the expression of Dhh and clinical stage, histologic subtype or survival has been reported, it is currently unclear whether expression of the Dhh ligand is associated with decreased survival [20]. Other analyses of ovarian carcinoma samples have suggested that elevated Gli1 protein expression is an independent factor associated with decreased survival when adjusting for age, stage, grade and histologic type [18].

*In vitro* and limited *in vivo* studies provide evidence to suggest that Hh signaling pathway plays an important role in ovarian cancer cell proliferation. Shh was detected in a number of ovarian cancer cell lines and addition of a monoclonal antibody against Shh resulted in a dose dependent decrease in cell proliferation [21]. Similarly, treatment of cultured ovarian cancer cells with the Smo inhibitor cyclopamine has been found to induce cell cycle arrest in G1 and promote apoptosis [20]. Moreover, ectopic expression of Gli1 in ovarian cancer cells resulted in increased cell proliferation, mobility and invasive properties [18]. Ovarian carcinoma cell line derived xenografts are also susceptible to Hh pathway inhibition as their growth is markedly impaired following treatment with cyclopamine [21]. The majority of available data suggest that an activated Hh signaling cascade may be a potential driver of neoplasia in a significant proportion of epithelial ovarian cancers making it an attractive target for therapeutic inhibition.

We have explored this possibility using the Hh pathway inhibitor IPI-926, a derivative of cyclopamine with increased oral bioavailability and a longer half-life [22]. IPI-926 antagonizes the Hh pathway by inhibiting Smo. IPI-926 induces a dose dependent reduction in *Gli1* mRNA expression and durable tumor remission in a murine medulloblastoma model [22]. Although IPI-926 had no effect as a single agent in a mouse model of small cell lung cancer prior to chemotherapy, animals treated with IPI-926 following chemotherapy exhibited a dramatic inhibition in tumor re-growth [22]. Given these pre-clinical data, we sought to determine whether inhibition of the Hh pathway by cyclopamine or IPI-926 had an effect on the growth of human papillary serous ovarian cancer xenografts derived from primary human tissue obtained at the time of initial surgery. We then assessed the anti-tumor activity of IPI-926 in our xenograft model in combination with paclitaxel and carboplatinum and as a single agent maintenance therapy. Finally, the effect of a single dose and prolonged IPI-926 treatment on Hh pathway activity was determined in both the stromal and tumor cell populations. Our results support the hypothesis that the Hh signaling pathway contributes to the pathogenesis of serous ovarian cancer and inhibition of this pathway may have therapeutic benefit.

## Materials and Methods

### Immunohistochemical analysis

An ovarian cancer tissue microarray (T8235725, Biochain, Hayward, CA) comprising 64 benign and malignant ovarian human tumors was used for immunohistochemical analysis of Shh protein expression. Antigen retrieval was carried out in citrate target retrieval buffer using a pressure cooker for 40 minutes and the sections were treated with 3% hydrogen peroxide and incubated in Background Sniper (Biocare, Concord, CA) to block any non-specific binding. Incubation of the sections with the anti-Shh monoclonal antibody ab53281 (Abcam, Cambridge, MA) diluted 1∶2000 in DaVinci Green diluent (Biocare Medical, Concord, CA) was carried out at room temperature for 90 minutes. The sections were then incubated with rabbit on rodent polymer system (Biocare Medical, Concord, CA) for 45 minutes at room temperature. Slides were then treated with 3, 3′-diaminobenzidine chromagen (DAB) for 5 minutes for visualization. Sections were then counterstained with hematoxylin, dehydrated and mounted. Total rabbit IgG substituted for the primary antibodies served as a negative control.

### Tumor collection and propagation *in vivo*

Excess human tumor or ascites samples were obtained through an IRB approved centralized banking infrastructure at the Massachusetts General Hospital (MGH). Written informed consent is received from all participants. There were no samples derived from minors in this study. The MGH Gyn tissue repository protocol (07-049) was reviewed and approved by the Dana-Farber Harvard Cancer Center (DFHCC). In addition, we utilized archived samples derived from MGH pathology under an institutional approved protocol (2008P001695).

Single cell suspensions from either enzymatically processed solid tumor tissue or ascites were then depleted of hematologic components as described [23]. A specified number of cells were suspended in PBS∶Matrigel® (1∶1) and injected subcutaneously (s.c.) into 6–8 week old female NOD/SCID mice (Jackson Laboratory, Bar Harbor, ME). Animals were monitored regularly to determine tumor formation and euthanized when they became moribund or had excessive tumor burden. All animal experiments were conducted in strict accordance with the recommendations in the Guide for the Care and Use of Laboratory Animals of the National Institutes of Health. The protocol was approved by the Massachusetts General Hospital Sub-Committee on Research and Animal Care (protocol number 2005N000273). Every effort was made to minimize suffering.

For continued propagation in mice, the xenograft tumors were excised and enzymatically processed to a single cell suspension. The suspension was depleted of mouse H-2K^d+^ cells using MACS beads (Miltenyi Biotech, Bergisch Gladbach, Germany) and the remaining tumor-derived cells were re-injected subcutaneously into new recipient NOD/SCID mice. All of the primary human papillary serous ovarian tumors utilized in this study had undergone 4–5 passages *in vivo* and the serous histology of each was maintained over the serial transplantation process. Animals were housed and maintained in accordance with institutional guidelines.

### Treatment of mice with Hh pathway inhibitors

#### Cyclopamine

Mice bearing matched sized tumors (300–600 mm^3^) from the human serous ovarian cancer sample SOC11 were randomized into two groups of six mice. Each cohort received either 25 mg/kg cyclopamine or vehicle (30% (w/v) 2-hydroxypropyl-β-cyclodextrin (HPBCD) in 0.1 M sodium citrate phosphate buffer, pH 3.0) by daily oral gavage. The treatment period spanned 13 days. Tumor measurements and animal weights were assessed every 4 days. Tumors were measured weekly with calipers and tumor volume (mm^3^) was determined using the formula [length (mm)×width (mm)×width (mm)]/2. Mouse weights were assessed every 4–7 days to monitor potential toxicity associated with each treatment. At the end of the treatment period the animals were euthanized and the tumors were harvested for further analysis.

*IPI-926:* Twenty-four mice bearing SOC12, SOC13 or SOC14 xenograft tumors were randomized into four cohorts with matched tumor volumes (300–600 mm^3^). The paclitaxel/carboplatinum (T/C) alone cohort received weekly intraperitoneal (i.p.) injection of 15 mg/kg paclitaxel and 50 mg/kg carboplatinum and the IPI-926 vehicle 5% (w/v) (HPBCD) administered every other day by oral gavage. The IPI-926 alone cohort received 40 mg/kg IPI-926 every other day by oral gavage and Cremophor∶ethanol (1∶1, paclitaxel vehicle) and saline (carboplatinum vehicle) by weekly i.p. injection. Mice in the T/C+IPI-926 cohort received weekly paclitaxel (15 mg/kg, i.p.) and carboplatinum (50 mg/kg, i.p.) and 40 mg/kg IPI-926 (every other day, oral gavage). The control cohort received the paclitaxel, carboplatinum and IPI-926 vehicles at the dosing schedule appropriate for each. Tumor volumes and mouse weights were assessed every 4–7 days as described above.

The last dose in the T/C cohort was administered on day 14. After 21 days, mice in the control and IPI-926 alone cohorts were euthanized and their tumors were harvested. Portions of each tumor were snap frozen for RNA analysis and embedded in paraffin for histological analysis.

Following the completion of T/C treatment, mice in the T/C alone and T/C+IPI-926 cohorts continued to receive either IPI-926 vehicle (T/C alone group) or 40 mg/kg IPI-926 (T/C+IPI-926 group) every other day by oral gavage. This maintenance treatment was carried out for 18–30 days. At the end of the maintenance period, animals were euthanized and tumors were harvested and processed for RNA and histological analyses as described above.

### Acute IPI-926 treatment

Mice harboring xenografts (300–600 m^3^) derived from SOC12, SOC13 or SOC14 were treated with either 40 mg/kg IPI-926 or IPI-926 vehicle by oral gavage. After 24 hours the animals were euthanized. Their tumors were harvested and portions of each were snap frozen for RNA analysis and embedded in paraffin.

### RT-PCR analysis

RNA was isolated from frozen tumor tissue using the GenElute™ mammalian RNA extraction kit (Sigma Aldrich, St. Louis, MO) following the manufacturer's instructions. Two micrograms of isolated RNA were used to synthesize cDNA using the Invitrogen Superscript ™ VILO kit (Invitrogen, Carlsbad, CA). Primers and probes for mouse *Gli1* were purchased from Applied Biosystems Inc. (Carlsbad, CA, catalog number Mm00494645_m1). Primers and probes for human *Gli1* were designed in-house: forward: 5′-CTTTCATCAACTCGCGATGC-3′; reverse: 5′-GCTCATGGTGCCAATGGAG-3′; probe: 5′FAM-CATCTCCAGGAGGCTCCTACGGTCA TC- TAMRA-3′. Primers were designed to span exon-exon junctions to prevent detection of genomic DNA. The expression levels of the human and mouse *Gli1* genes were normalized to GAPDH and quantified as described by Applied Biosystems Inc.

In addition to *Gli1* mRNA, we analyzed *SHH*, *SMO*, *and Ptch1* mRNA levels in the 4 patient samples used to generate tumor explants for testing the inhibition of the Hhog pathway. Primers and probes for human *SHH* (catalog number Hs00179843_m1), *SMO* (catalog number Hs00170665_m1), and *Ptch1* (catalog number Hs00970980_m1) were purchased from Applied Biosystems Inc. (Carlsbad, CA).

The expression levels of the human *SHH*, *SMO*, *and Ptch1* genes were normalized to GAPDH and quantified as described by Applied Biosystems Inc.

### Analysis of *Gli1* expression in human ovarian tumor stroma

High-grade late-stage serous primary ovarian tumors specimens were obtained prior to chemotherapy from 19 ovarian cancer patients hospitalized at the Brigham and Women's Hospital between 1990 and 2000. Classification was determined according to the International Federation of Gynecology and Obstetrics standards. All specimens and their corresponding clinical information were obtained under IRB-approved protocols. Microdissection and RNA isolation were performed as previously described [24]. Briefly, fibroblasts from 7 µm frozen sections were microdissected using a MD LMD laser microdissecting microscope (Leica, Wetzlar, Germany) and lysed in RLT lysis buffer (Qiagen, Valencia, CA). RNA was extracted and purified using the RNeasy Micro kit (Qiagen, Valencia, CA). Total RNA specimens were quantified and checked for quality with a Bioanalyzer 2100 system (Agilent, Palo Alto, CA) and then amplified as previously described [24]. Quantitative real-time PCR (qRT-PCR) was performed on 50 ng of double-amplified product from the 19 specimens using primer sets specific for *Gli1* (forward ACCCCCTGGACTCTCTTGAT, reverse GACACTCATGTTGCCCACAG). An iCycler iQ Real-time PCR Detection System (Bio-Rad Laboratories, Hercules, CA) was used in conjunction with the SuperScript III Platinum SYBR Green One-Step qRT-PCR kit (Invitrogen, Carlsbad, CA) according to the manufacturer's instructions.

### Statistical Analysis

Non-parametric Wilcoxan rank sum tests for unpaired samples were used to compare tumor sizes in the cyclopamine and IPI-926 treatment experiments. Student's t-tests were used to determine the statistical significance of results obtained in the IPI-926 acute and prolonged treatment experiment. Significance was set at p≤0.05. STATA (College Station, TX) v10 software was used. The survival analysis was performed using GraphPad Prism version 5.00 for Windows (GraphPad Software, San Diego California USA).

## Results

### Shh and Gli1 expression in human ovarian tumors

**Figure 1:**
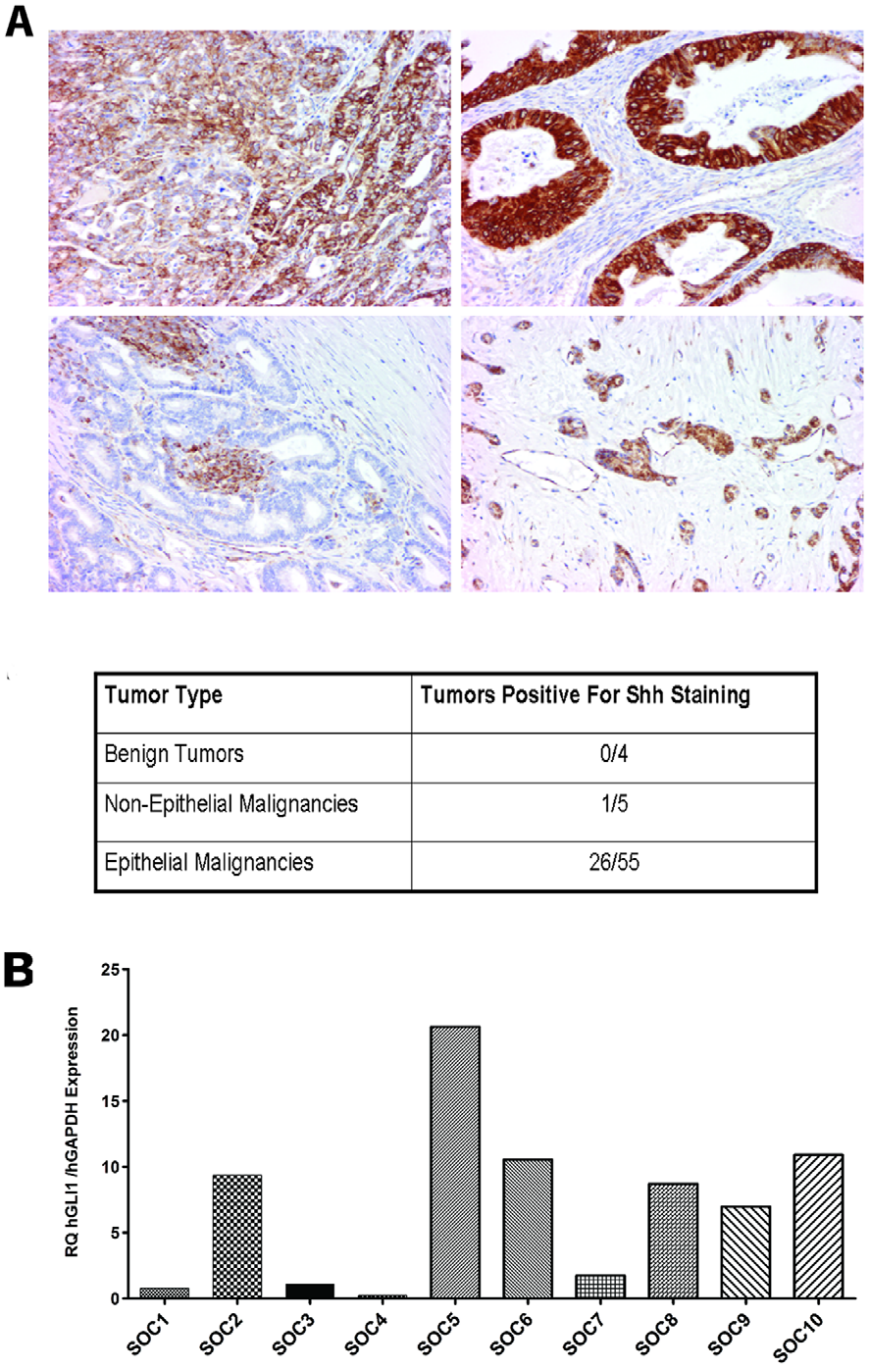
Sonic hedgehog (Shh) and Gli1 are variably expressed in human ovarian tumors. A. Immunohistochemical analysis of Shh expression was carried out on a tissue microarray carrying 64 individual primary ovarian tumors to assess both the frequency and level of Shh expression in human ovarian cancer. The Shh staining in a poorly differentiated serous cystadenocarcinoma (upper left panel), a poorly differentiated mucinous cystadenocarcinoma (upper right panel), a well-differentiated cystadenocarcinoma (lower left panel), and a poorly differentiated adenocarcinoma (lower right panel) is shown. The table summarizes the frequency of Shh staining in the benign tumors and non-epithelial and epithelial malignancies present in the ovarian tumor microarray. B. Real-time Q-PCR was performed for analysis of *Gli1* mRNA levels in 10 primary human papillary serous ovarian cancer tumors. Primers specific for human *Gli1* and *GAPDH* were used. For each sample, the level of *Gli1* mRNA relative to the level of *GAPDH* mRNA is shown.

**Figure 2:**
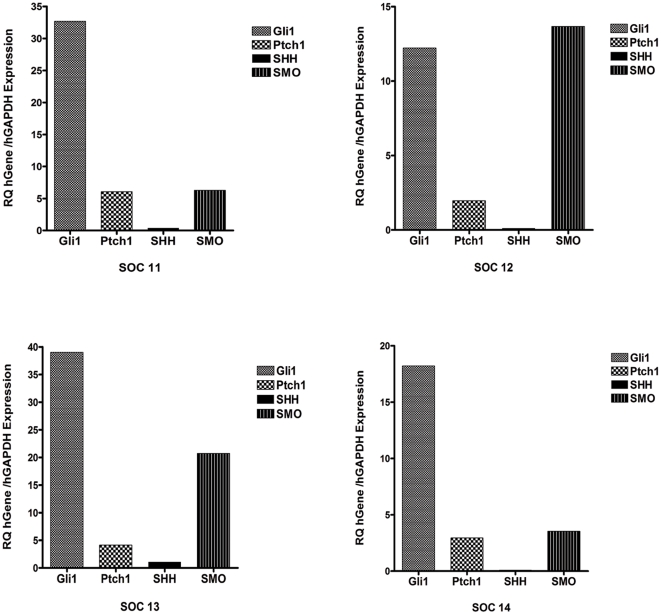
Hedgehog pathway components are differentially expressed in primary human ovarian tumors. The expression of *Gli1*, *Ptch1*, *SHH* and *SMO* was analyzed by RT-qPCR in 4 primary human serous ovarian tumors (SOC11-SOC14) established as xenografts in NOD/SCID mice. Primers specific for each human gene and human *GADPH* were used. For each tumor, the levels of each gene relative to *GADPH* mRNA are shown.

Recent studies have highlighted the role of the Hh signaling pathway in development and progression of solid tumors [25]. We assessed the relative expression of the Hh pathway members Shh and Gli1 in a subset of ovarian tumors. As shown in Figure 1A, immuohistochemical analysis of Shh expression on a tissue microarray comprising benign and malignant tumors determined that 47% of the evaluated malignant epithelial ovarian tumors had detectable levels of Shh expression. In comparison, Shh expression was detected in only 1 of 5 non-epithelial ovarian tumors. As evidenced by the examples shown in Figure 1A, both the level and distribution of Shh expression varied widely across the analyzed tumors. We then examined the expression of *Gli1* mRNA in the individual primary human papillary serous ovarian tumors SOC1-SOC10 (see Table S1 for patient details) by RT-PCR. We observed a wide range of *Gli1* mRNA expression across the analyzed tumors (Figure 1B). In addition, we analyzed *SHH*, *SMO*, *Ptch1*, *and Gli1* mRNA levels in the 4 individual patient samples we used to generate tumor xenografts for analyzing the effect of Hh pathway inhibition on ovarian tumor growth *in vivo* (Figure 2). In each of the tumors, *Gli1* and *SMO* were more abundantly expressed relative to *Ptch1*. There was little to no *SHH* mRNA expressed in these samples. Taken together, these expression analyses suggest that the Hh signaling pathway is activated in a subset of human ovarian tumors as previously reported.

### Inhibition of Hh signaling blocks the growth of human serous ovarian tumor xenografts

**Figure 3:**
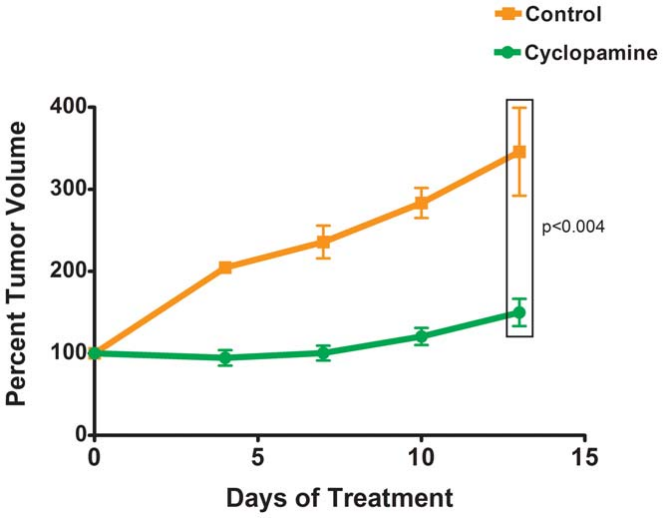
Cyclopamine alone impedes ovarian tumor growth. Mice bearing human papillary serous ovarian tumor xenografts from sample SOC11 were randomized into 2 cohorts with matched tumor volumes and treated with either vehicle or cyclopamine. Tumor volumes were assessed regularly. Change in tumor volume is shown relative to the tumor volume at the beginning of the experiment. Error bars represent the standard error of the mean (S.E.M). The significance was determined using a Wilcoxon rank sum test.

To assess the functional significance of the Hh pathway in the progression of ovarian cancer, we treated mice harboring human serous ovarian tumor xenografts generated from the platinum sensitive tumor SOC11 with either the Hh pathway inhibitor cyclopamine or vehicle and regularly assessed the effect of each treatment on tumor volume. In the cyclopamine-treated mice, tumor growth was precluded over the thirteen-day treatment period resulting in a significantly (p<0.004) decreased tumor volume at day 13 relative to that observed in the vehicle treated animals (Figure 3). Minimal toxicity was observed in cyclopamine treated animals with no observed statistically different changes in mouse weight (Figure S1).

**Figure 4:**
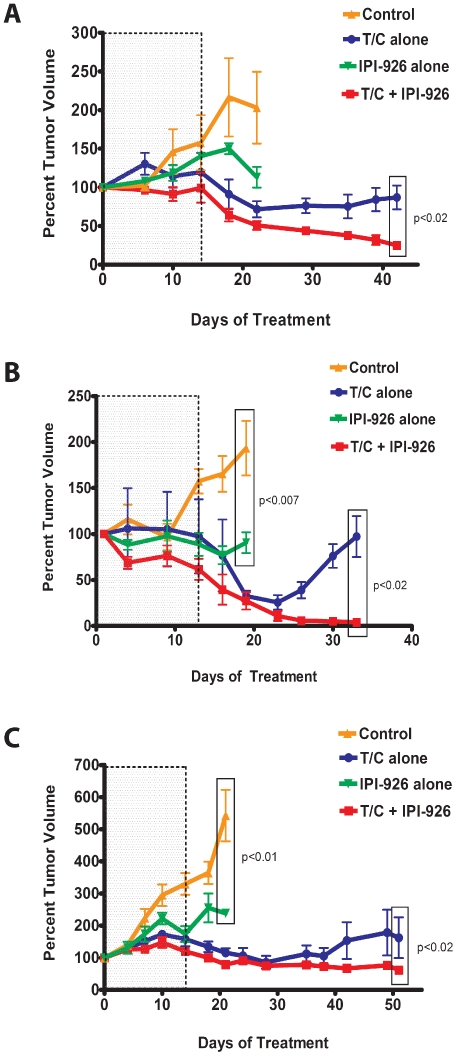
IPI-926 is an effective maintenance therapy. Mice bearing tumors derived from patients diagnosed with serous ovarian cancer were randomized into 4 cohorts (n = 4–6 animals per treatment cohort) with matched tumor volumes and subjected to the indicated treatment regimens. Three independent papillary serous ovarian cancer samples, SOC12 (A), SOC13 (B) and SOC14 (C) were used as biological replicates. Cohorts were initially treated with vehicle alone (Control), paclitaxel and carboplatinum (T/C) alone, IPI-926 alone or T/C+IPI-926 as indicated. The last T/C dose was delivered on day 14 (indicated by the dotted line). Percent change in tumor volume represents the change in tumor volume relative to the tumor volume at the beginning of the experiment. Error bars represent the standard error of the mean (S.E.M). Significance was determined using a Wilcoxon rank sum test.

Our analysis of cyclopamine effect on tumor xenograft growth suggested that inhibition of Hh signaling may be an effective approach to controlling ovarian tumor progression. We next carried out similar experiments using the more chemically potent and clinically applicable Hh pathway inhibitor IPI-926 currently in Phase I/II clinical trials in non-ovarian solid tumors. IPI-926 is derived from cyclopamine and has improved pharmaceutical properties including higher potency, a longer half-life and increased bioavailability. We were especially interested in examining the potential utility of IPI-926 in combination with standard first line chemotherapy. We carried out three separate experiments utilizing independent primary human serous ovarian xenograft tumors (SOC12, SOC13, and SOC14) to analyze the anti-tumor activity of IPI-926 alone or in combination with paclitaxel and carboplatinum (T/C). The tumor xenografts were derived from 2 patients with platinum resistant disease (SOC12, SOC13) and 1 patient with platinum sensitive disease (SOC14) and the results of those analyses are shown in Figure 4. Mice bearing primary human serous ovarian tumor xenografts were randomized into four treatment groups which received either vehicle control, IPI-926 alone, T/C alone or IPI-926 and T/C. Over the initial treatment period (days 0–21), tumor volume in mice treated with the vehicle control increased dramatically (Figures 4A, 4B, 4C). Treatment of SOC12, SOC13 and SOC14 xenografts with T/C alone resulted in a significant decrease in tumor volume (p<0.05; p<0.007; p<0.001 respectively) relative to the control treatment. Like cyclopamine, IPI-926 as a single agent had a statistically significant inhibitory effect on tumor growth relative to vehicle control for both SOC13 (Figure 4B, p<0.007) and SOC14 (Figure 4C, p<0.01). The combination of IPI-926 and T/C demonstrated no greater anti-tumor activity than T/C alone for all tested tumors. In these analyses, we observed no significant weight loss in animals treated with IPI-926 as a single agent compared to vehicle treated animals (Figure S2).

We next examined the anti-tumor activity of IPI-926 following cessation of T/C treatment. For these analyses, those mice receiving either T/C alone or in combination with IPI-926 were administered their third and final weekly dose of T/C on day 14 of the treatment regimen (dotted line, Figure 4). The animals were then maintained on either vehicle (T/C alone treated cohort) or IPI-926 (T/C+IPI-926 treated cohort) and tumor volumes were assessed at regular intervals. As shown in Figures 4A, 4B and 4C, continued administration of IPI-926 following completion of T/C treatment maintained tumor regression, and in one case (SOC13, Figure 4B), resulted in a further decrease in tumor volume to levels that were virtually undetectable. In contrast, we observed steadily increasing tumor growth in mice treated with vehicle following the cessation of T/C administration. Thus, continued IPI-926 treatment resulted in a significant (p<0.02) difference in tumor volume relative to that observed in vehicle treated mice for all three individual primary tumors.

### IPI-926 inhibits Hh pathway activity in both the stroma and tumor

**Figure 5:**
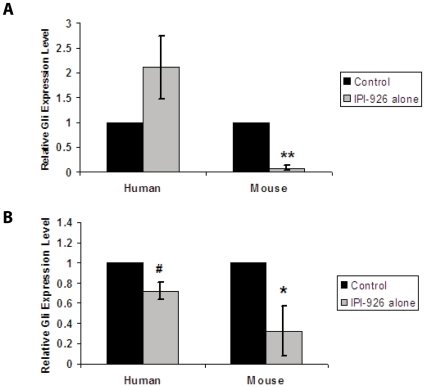
IPI-926 blocks Hh pathway activity in mouse stromal and human tumor cells. *Gli1* mRNA expression levels in xenograft tumors derived from SOC12, SOC13, and SOC14 were analyzed by real time quantitative PCR following treatment of mice with IPI-926 for 24 hours (upper panel) or 21 days (lower panel). The relative levels of human (left panel) and mouse (right panel) *Gli1* mRNA expression were analyzed using species-specific primers. Error bars represent the standard error of the mean (S.E.M). The significance (#p≤0.03, *p = 0.05, ** p≤0.001) was determined using Student's t –test.

Recent data have suggested that Hh signaling in the stroma is required for tumor development in mouse xenograft models of pancreatic and colon cancer (25). To assess the site of action of IPI-926 in our system, we analyzed *Gli1* mRNA expression in both mouse stroma and human tumor cells following either acute or prolonged treatment with IPI-926. For the acute studies, mice harboring xenografts generated from SOC12, SOC13 and SOC14 were treated with either vehicle or IPI-926 for 24 hours. Following tumor harvest, the relative expression of mouse and human *Gli1* was assessed by RT-PCR using species-specific primers. The acute IPI-926 treatment led to a rapid significant decrease in mouse *Gli1* with no inhibition of human *Gli1* expression (Figure 5, upper panel). We also analyzed stromal and tumor specific *Gli1* expression in the SOC12, SOC13 and SOC14 xenografts harvested from the mice treated with either vehicle or IPI-926 for 21 days in the studies shown in Figure 4. Like in the acute setting, prolonged IPI-926 treatment led to a significant inhibition of *Gli1* mRNA expression in the stromal (p<0.05) compartment (Figure 5, lower panel). In contrast to what was also observed in the acute setting, moderate inhibition of *Gli1* mRNA expression was observed in the tumor (p<0.03) following prolonged treatment.

### High *Gli1* expression in the stroma of human papillary serous ovarian tumors correlates with poor survival

**Figure 6:**
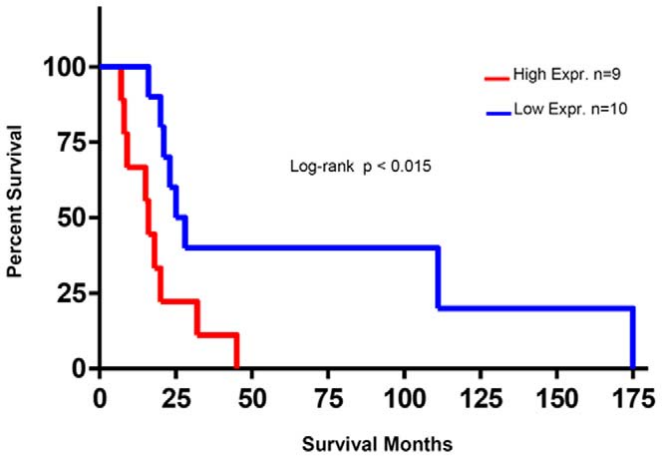
Stromal levels of *Gli1* mRNA correlate with survival. Survival analysis data obtained for *Gli1* mRNA expression on microdissected stroma samples of 19 patients using qRT-PCR validation. Higher *Gli1* mRNA expression correlated with decreased survival (Log-rank Test, p<0.015) with a median survival of 26.50 months for patients with low *Gli1* mRNA expression versus 16 months for the patients that had tumors with higher *Gli1* mRNA expression in stroma.

In order to validate that *Gli1* mRNA is expressed in the stroma of human serous ovarian tumors, we examined the relative expression of *Gli1* in stroma microdissected from a second cohort of 19 human primary ovarian serous tumors (Table S2). The micro-dissected stroma of these tumors demonstrated a full range of *Gli1* mRNA expression (data not shown). Univariate survival analysis revealed that high *Gli1* mRNA expression in the stroma correlated with a significantly worsened survival (Figure 6).

## Discussion

Numerous investigators have documented the role of the Hh signaling pathway in the development and maintenance of solid tumors [4], [6], [7], [8], [9], [10], [11], [12]. Recent evidence suggests that activation of this pathway is involved in the pathogenesis of ovarian cancer (18, 20, 21). In our study, therapeutic targeting of this pathway led to inhibition of human papillary serous tumor growth. The Hh pathway inhibitor IPI-926 demonstrated significant anti-tumor activity when administered as both a single agent and after conventional cytotoxic therapy in primary tumor xenografts and mediated its effects in both stromal and epithelial cells.

We initiated our investigation by analyzing the expression of both the Hh pathway ligand Shh by immunohistochemistry and the *Gli1* transcription factor by real-time PCR in human ovarian tumors. While some tumors, such as the majority of basal cell carcinomas, have specific genetic defects that constitutively activate the Hh pathway driving tumor growth, other solid tumors appear to rely on increased levels of ligand to drive the Hh signaling cascade [6], [25]. Shh expression was previously reported to be evident in a majority of ovarian cancer samples [18], although this finding was disputed by Yang and colleagues [19] who detected Shh expression in approximately 32% of samples. We observed expression of Shh in 47% of the evaluated epithelial ovarian malignancies. In contrast, Shh expression was absent in benign ovarian tumors and less detectable in non-epithelial ovarian malignancies. Our findings support previous work suggesting that increased Hh pathway expression can contribute to the pathogenesis of papillary serous ovarian carcinoma.

Upon activation of the Hh signaling pathway, the Gli1 transcription factor translocates to the nucleus and induces both the expression of a number of genes involved in cell proliferation and differentiation and its own transcription [26]. siRNA-mediated knockdown of Gli1 has been shown to reduce cell proliferation rates [20]. Previous studies have reported elevated Gli1 expression in ovarian cancer cell lines and primary human ovarian tumor samples (18, 20). We analyzed the level of *Gli1* mRNA by real-time PCR in 10 individual primary human papillary serous ovarian tumors and detected a wide variability in expression. We also evaluated *SHH*, *SMO*, *Ptch1* and *Gli1* expression in the 4 additional patient tumors used to generate xenografts for our *in vivo* analyses. In each of these tumors, *Gli1* and *SMO* mRNA were expressed at a higher level than either *Ptch1* or *SHH* transcripts. Collectively, our results suggest that the Hh signaling pathway may be active in a subset of primary high grade serous ovarian tumors. Women whose tumors manifest Hh pathway activation may be candidates for potentially more effective targeted therapy directed against this pathway.

Pre-clinical studies have reported that tumor growth was significantly reduced in immunocompromised mice harboring tumors generated from established ovarian cancer cell lines following treatment with the naturally occurring Hh pathway inhibitor cyclopamine [20]. In this study, we utilized primary papillary serous ovarian carcinoma cells from multiple patients to generate xenograft tumors in NOD/SCID mice and evaluate the effect of Hh pathway inhibition on tumor growth. In our model, cyclopamine demonstrated significant single agent activity. To extend these findings, we carried out similar analyses using the semi-synthetic cyclopamine derivative IPI-926 with increased potency against the target (22). Like cyclopamine, IPI-926 demonstrated anti-tumor activity against two of the three analyzed human papillary serous ovarian tumor xenografts when administered as a single agent. Combined IPI-926 and T/C treatment demonstrated no enhanced anti-tumor activity than treatment with T/C alone. To assess the therapeutic potential of IPI-926 further, we evaluated its efficacy as a maintenance therapy. In these analyses, T/C treatment was stopped after administration of three doses, and the effect of continued IPI-926 treatment relative to vehicle control treatment on tumor growth was evaluated. As expected, the removal of T/C resulted in tumor re-growth in the presence of vehicle. In contrast, continued IPI-926 treatment inhibited ovarian tumor resurgence in all of the analyzed xenografts following the completion of conventional cytotoxic chemotherapy.

Previous studies have determined that Hh pathway inhibition of tumor growth in pancreatic and colon carcinoma xenografts models blocks paracrine signaling between the stroma and tumor [27]. We examined the effect of acute and prolonged IPI-926 treatment on stromal and tumor *Gli1* expression in our ovarian xenograft model. Our results show that *Gli1* expression in the stroma was specifically and significantly reduced within 24 hours of IPI-926 treatment while expression of *Gli1* in tumor cells was unaffected. However, prolonged (21 days) IPI-926 treatment resulted in significant decreases in both stromal and tumor *Gli1* expression. These data suggest prolonged inhibition of stromal Hh signaling could ultimately lead to decreased Hh signaling in tumor cells. Though the stromal compartment in this model is murine, the *Gli1* mRNA expression data from the microdissected stroma of human serous ovarian cancer confirms that the Hh pathway is active in human ovarian stroma, and that the degree of activation may be of prognostic value. In this cohort, those with elevated *Gli1* mRNA levels had worse overall survival, suggesting that targeting the Hh cascade would be of greatest benefit to those who require meaningful intervention.

In conclusion, our study suggests that the Hh pathway has a role in the pathogenesis of papillary serous ovarian cancer that may be mediated by both stromal and intra-tumor *Gli1* levels. IPI-926 demonstrated anti-tumor activity as both a single agent and as a maintenance therapy after conventional cytotoxic therapy in our human ovarian cancer xenograft models. Though further investigation will be required to determine precisely how Hh inhibition exerts this significant anti-neoplastic effect, our data indicate that therapies for ovarian cancer directed against the Hh cascade hold promise and merit exploration in clinical trials.

## Supporting Information

Figure S1 **Change in percent weight over the course of single agent cyclopamine therapy.** No statistical difference in weight was observed among the animals treated with cyclopamine and vehicle.
(TIF)

Figure S2 **Change in percent weight over the course of treatment with IPI-926 and paclitaxel/carboplatinum (T/C) therapy.** T/C consistently induced weight loss that was statistically different from control in 2 of the 3 experiments (A and C). No differences in weight were observed between vehicle and IPI-926 alone treated animals in all experiments.
(TIF)

Table S1 **Table summarizing clinical characteristics of the 14 patients whose serous ovarian cancer specimens were prospectively collected.**
(PPT)

Table S2 **Table summarizing the survival characteristics of the 19 patient cohort with micro-dissected serous ovarian tumors.**
(DOC)
