# Supplementary figures and images for: Inhibition of Hedgehog Signaling Antagonizes Serous Ovarian Cancer Growth in a Primary Xenograft Model
Source: PLoS One. 2011 Nov 29;6(11):e28077. doi: 10.1371/journal.pone.0028077 (PMC3226669; doi:10.1371/journal.pone.0028077)

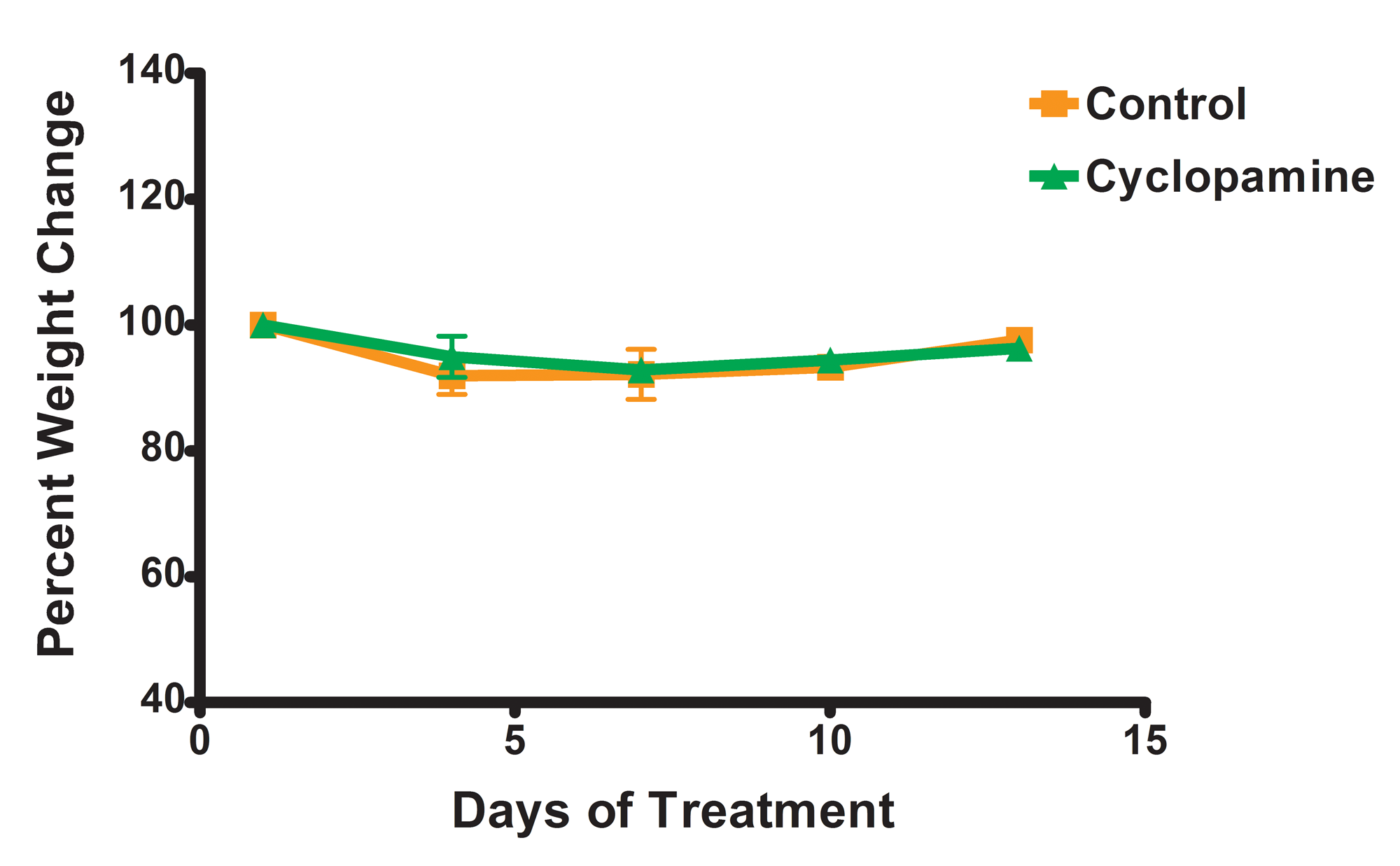

Supplement: Figure S1 — Change in percent weight over the course of single agent cyclopamine therapy. No statistical difference in weight was observed among the animals treated with cyclopamine and vehicle. (TIF) [file pone.0028077.s001.tif]

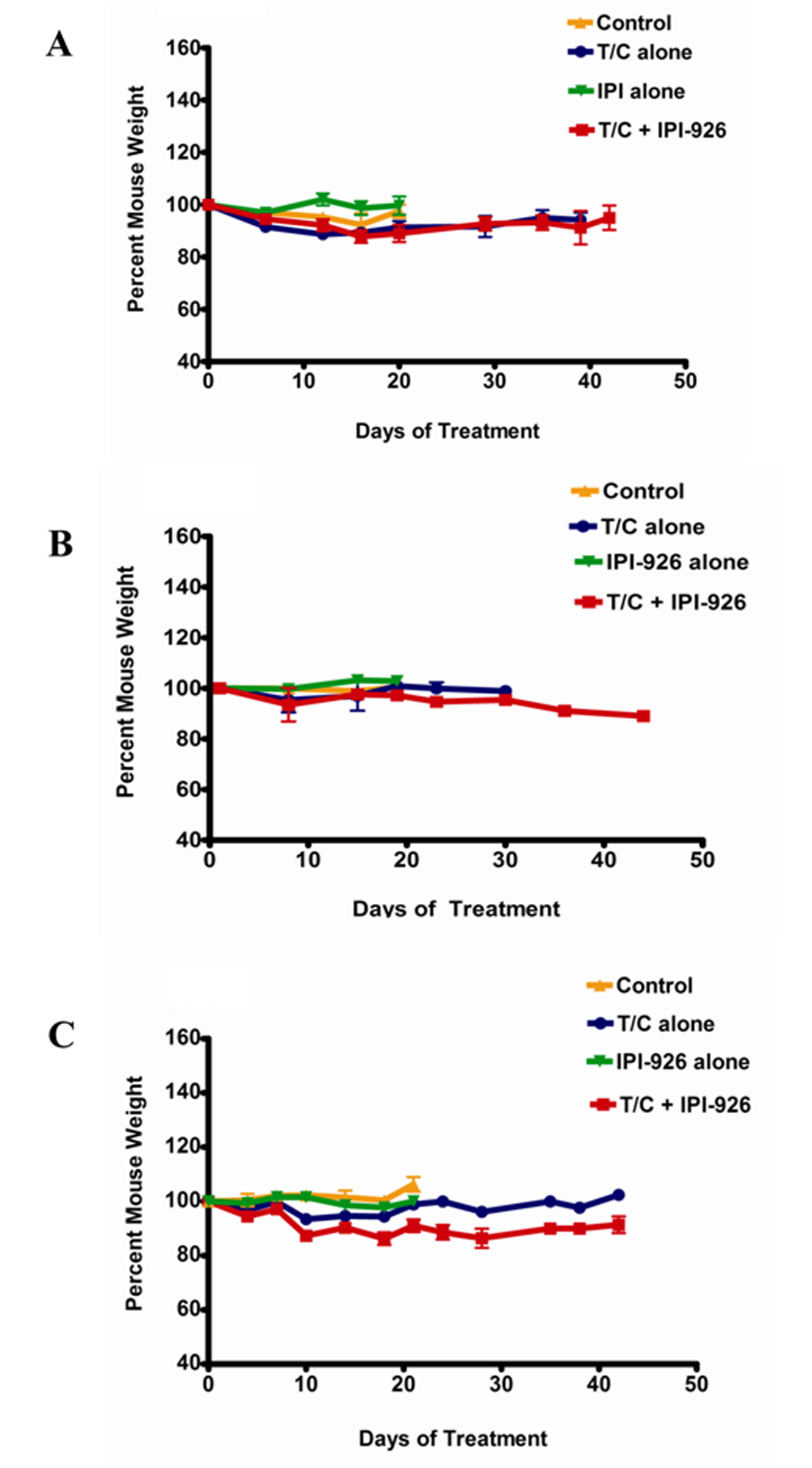

Supplement: Figure S2 — Change in percent weight over the course of treatment with IPI-926 and paclitaxel/carboplatinum (T/C) therapy. T/C consistently induced weight loss that was statistically different from control in 2 of the 3 experiments (A and C). No differences in weight were observed between vehicle and IPI-926 alone treated animals in all experiments. (TIF) [file pone.0028077.s002.tif]
