# Supplementary material for: Inhibition of Hedgehog Signaling Antagonizes Serous Ovarian Cancer Growth in a Primary Xenograft Model
Source: PLoS One. 2011 Nov 29;6(11):e28077. doi: 10.1371/journal.pone.0028077 (PMC3226669; doi:10.1371/journal.pone.0028077)
Supplement: Table S1 — Table summarizing clinical characteristics of the 14 patients whose serous ovarian cancer specimens were prospectively collected. (PPT) [file pone.0028077.s003.ppt]

## Slide 1
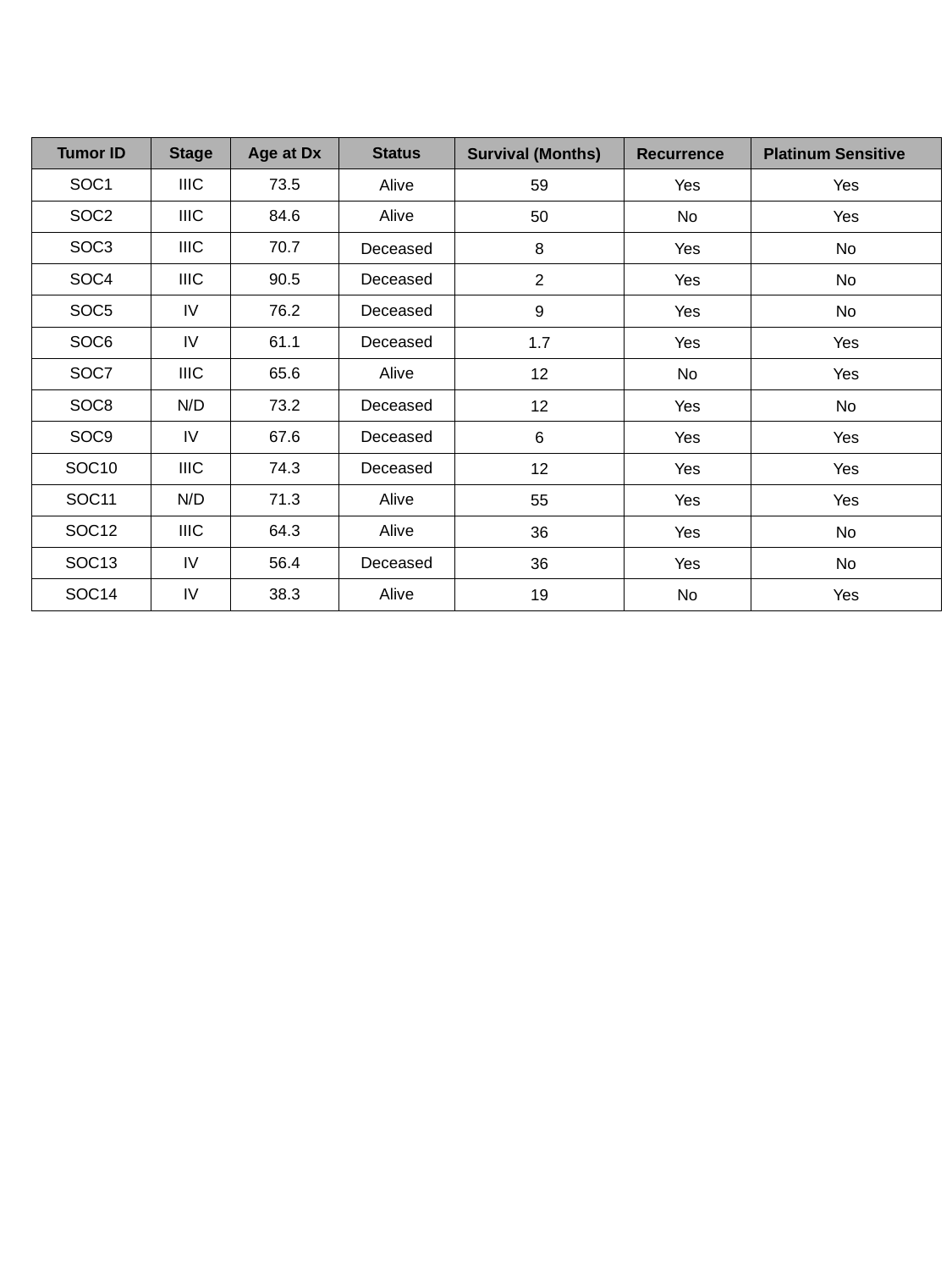

| Tumor ID | Stage | Age at Dx | Status | Survival (Months) | Recurrence | Platinum Sensitive |
| --- | --- | --- | --- | --- | --- | --- |
| SOC1 | IIIC | 73.5 | Alive | 59 | Yes | Yes |
| SOC2 | IIIC | 84.6 | Alive | 50 | No | Yes |
| SOC3 | IIIC | 70.7 | Deceased | 8 | Yes | No |
| SOC4 | IIIC | 90.5 | Deceased | 2 | Yes | No |
| SOC5 | IV | 76.2 | Deceased | 9 | Yes | No |
| SOC6 | IV | 61.1 | Deceased | 1.7 | Yes | Yes |
| SOC7 | IIIC | 65.6 | Alive | 12 | No | Yes |
| SOC8 | N/D | 73.2 | Deceased | 12 | Yes | No |
| SOC9 | IV | 67.6 | Deceased | 6 | Yes | Yes |
| SOC10 | IIIC | 74.3 | Deceased | 12 | Yes | Yes |
| SOC11 | N/D | 71.3 | Alive | 55 | Yes | Yes |
| SOC12 | IIIC | 64.3 | Alive | 36 | Yes | No |
| SOC13 | IV | 56.4 | Deceased | 36 | Yes | No |
| SOC14 | IV | 38.3 | Alive | 19 | No | Yes |
