# Supplementary material for: Inhibition of Hedgehog Signaling Antagonizes Serous Ovarian Cancer Growth in a Primary Xenograft Model
Source: PLoS One. 2011 Nov 29;6(11):e28077. doi: 10.1371/journal.pone.0028077 (PMC3226669; doi:10.1371/journal.pone.0028077)
Supplement: Table S2 — Table summarizing the survival characteristics of the 19 patient cohort with micro-dissected serous ovarian tumors. (DOC) [file pone.0028077.s004.doc]

| **Tumor ID** | **Age** | **Grade** | **Stage** | **Survival (Months)** | **Status** |
| --- | --- | --- | --- | --- | --- |
| 321 | 66 | 3 | IIIC | 45 | Deceased |
| 332 | 69 | 3 | IIIC | 7 | Deceased |
| 345 | 59 | 3 | IIIC | 18 | Deceased |
| 349 | 41 | 3 | IIIC | 175 | Deceased |
| 358 | 50 | 3 | IIIC | 21 | Deceased |
| 367 | 74 | 3 | IIIC | 16 | Deceased |
| 384 | 48 | 3 | IIIC | 20 | Deceased |
| 394 | 64 | 3 | IIIC | 16 | Deceased |
| 443 | 79 | 3 | IIIC | 111 | Deceased |
| 486 | 58 | 3 | IIIC | 28 | Deceased |
| 656 | 51 | 3 | IIIC | 25 | Deceased |
| 660 | 62 | 3 | IV | 20 | Deceased |
| 691 | 52 | 3 | IV | 32 | Deceased |
| 714 | 72 | 3 | IV | 8 | Deceased |
| 794 | 70 | 3 | IIIC | 15 | Deceased |
| 872 | 95 | 3 | IIIC | 9 | Deceased |
| 934 | 50 | 3 | IIIC | 58 | Alive |
| 1109 | 56 | 3 | IIIC | 34 | Alive |
| 1660 | 42 | 3 | IIIC | 23 | Deceased |
